# High-flow nasal cannula oxygen therapy in adults

**DOI:** 10.1186/s40560-015-0084-5

**Published:** 2015-03-31

**Authors:** Masaji Nishimura

**Affiliations:** Emergency and Critical Care Medicine, Tokushima University Graduate School, 3-18-15 Kuramoto, Tokushima, 770-8503 Japan

**Keywords:** Oxygen therapy, Physiological effects, Clinical trials, Anatomical dead space, PEEP effect

## Abstract

High-flow nasal cannula (HFNC) oxygen therapy comprises an air/oxygen blender, an active humidifier, a single heated circuit, and a nasal cannula. It delivers adequately heated and humidified medical gas at up to 60 L/min of flow and is considered to have a number of physiological effects: reduction of anatomical dead space, PEEP effect, constant fraction of inspired oxygen, and good humidification. While there have been no big randomized clinical trials, it has been gaining attention as an innovative respiratory support for critically ill patients.

Most of the available data has been published in the neonatal field. Evidence with critically ill adults are poor; however, physicians apply it to a variety of patients with diverse underlying diseases: hypoxemic respiratory failure, acute exacerbation of chronic obstructive pulmonary disease, post-extubation, pre-intubation oxygenation, sleep apnea, acute heart failure, patients with do-not-intubate order, and so on. Many published reports suggest that HFNC decreases breathing frequency and work of breathing and reduces needs of escalation of respiratory support in patients with diverse underlying diseases.

Some important issues remain to be resolved, such as its indication, timing of starting and stopping HFNC, and escalating treatment. Despite these issues, HFNC oxygen therapy is an innovative and effective modality for the early treatment of adults with respiratory failure with diverse underlying diseases.

## Introduction

**Figure 1:**
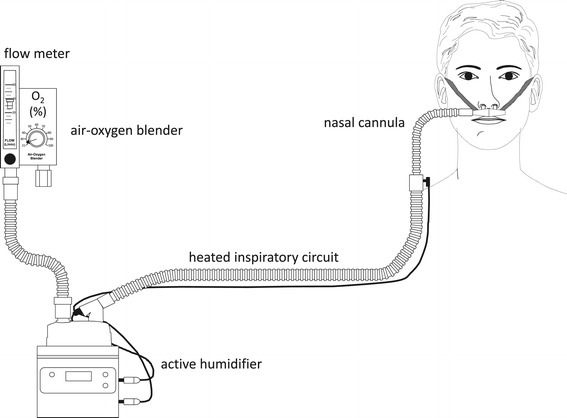
**Principle setup of high-flow nasal cannula oxygen therapy.** An air/oxygen blender, allowing from 0.21 to 1.0 F_I_O_2_, generates up to 60 L/min flow. The gas is heated and humidified through an active heated humidifier and delivered via a single-limb heated inspiratory circuit. The patient breathes the adequately heated and humidified medical gas through nasal cannulas with a large diameter.

The purpose of respiratory support is to maintain adequate ventilation and oxygenation. In this, ensuring adequate alveolar ventilation is essential for expelling carbon dioxide produced in the human body. Currently, to ensure adequate alveolar ventilation, minute ventilation is manipulated during invasive or noninvasive ventilatory support. For patients with acute exacerbation of chronic obstructive lung disease (COPD), noninvasive ventilation (NIV) has become the preferred primary modality for respiratory support because it enhances inspiratory tidal volume (V_T_) and maintains adequate alveolar ventilation [1]. Because of poor mask tolerance, however, NIV is sometimes inapplicable. High-flow nasal cannula (HFNC) oxygen delivery has been gaining attention as an alternative means of respiratory support for critically ill patients. The apparatus comprises an air/oxygen blender, an active heated humidifier, a single heated circuit, and a nasal cannula. At the air/oxygen blender, the inspiratory fraction of oxygen (F_I_O_2_) is set from 0.21 to 1.0 in a flow of up to 60 L/min. The gas is heated and humidified with the active humidifier and delivered through the heated circuit (Figure 1). Another major difference between NIV and HFNC is the interface. While interfaces for NIV increase anatomical dead space, those for HFNC actually decrease dead space. Since neither inspiratory push nor expiratory pull is effective in such an open circuit, HFNC cannot actively enhance V_T_. Even so, it helps COPD patients mainly by decreasing anatomical dead space and secondarily by improving alveolar ventilation.

Administration of supplemental oxygen has been the first-line therapy for hypoxemic patients. Oxygen is generally provided via face masks and nasal cannula. Several drawbacks are associated with these devices, which may limit efficacy and tolerance of oxygen delivery. Usually, oxygen is not humidified at low flow, and complaints, especially dry nose, dry throat, and nasal pain, are common. Bubble humidifiers are commonly used for humidifying air delivered to spontaneously breathing patients, but when absolute humidity is low, patients still complain of discomfort [2,3]. Insufficient heating and humidification leads to poor tolerance to oxygen therapy. Using conventional devices, oxygen flow is limited to no more than 15 L/min. Meanwhile, the inspiratory flow of patients with respiratory failure varies widely in a range from 30 to more than 100 L/min. The difference between patient inspiratory flow and delivered flow is large, and as a result, F_I_O_2_ is both inconstant and often lower than expected. As an alternative to conventional oxygen delivery for hypoxemic patients, HFNC oxygen therapy has been receiving more and more attention.

Most of the available data from this technique has been published in the neonatal field where it is increasingly used [4,5]. HFNC is considered to have a number of advantages over conventional oxygen delivery systems, resulting in better physiological effects. Recently, its use with critically ill adults has been dramatically rising. It has been applied to a variety of patients with diverse underlying diseases. While many of the studies have been clinical trials, no results from reliable, large, controlled clinical trials have yet been published. In the literature, the technique has also been called mini-CPAP (continuous positive airway pressure), transnasal insufflation, nasal high flow, nasal high-flow ventilation, high-flow therapy, and high-flow nasal cannula oxygen therapy. Here, we use the term HFNC throughout the text, in which we summarize the physiological effects of HFNC and then review the clinical trials.

## Review

### Physiological effect

Gas from an air/oxygen blender that can generate a total flow of up to 60 L/min is heated and humidified with an active humidifier and subsequently delivered through a heated circuit. High flow of adequately heated and humidified gas is considered to have a number of physiological effects.

1. High flow washes out carbon dioxide in anatomical dead space.
2. Although delivered through an open system, high flow overcomes resistance against expiratory flow and creates positive nasopharyngeal pressure. While the pressure is relatively low compared with closed systems, it is considered adequate to increase lung volume or recruit collapsed alveoli.
3. The difference between the inspiratory flow of patients and delivered flow is small and F_I_O_2_ remains relatively constant.
4. Because gas is generally warmed to 37°C and completely humidified, mucociliary functions remain good and little discomfort is reported.

### Anatomical dead space washout

Itagaki et al. have evaluated thoracoabdominal synchrony with respiratory inductance plethysmography [6]. They found that thoracoabdominal synchrony is better with HFNC than with face mask delivery. Breathing frequency is lower with HFNC, while PaCO_2_ and V_T_ (calculated from rib cage and abdominal measurements) remain constant. Since V_T_ is constant and breathing frequency is reduced, minute ventilation is lower. It is also likely that alveolar ventilation, along with PaCO_2_, is constant. This evidence suggests that there is less dead space. Lower breathing frequency with HFNC than with low-flow oxygen delivery has also been reported in other studies [7-10]. In a lung-injured-animal model, PaCO_2_ decreased as HFNC flow increased, and greater escape of gas more effectively decreased PaCO_2_. These results suggest effective carbon dioxide washout with HFNC [11]. Wettstein et al. compared F_I_O_2_ in healthy volunteers breathing with mouths opened and closed [12]. F_I_O_2_ was higher with mouth-open breathing. This may have been due to the reservoir function of the nose, the pharynx, and, potentially, the oral cavity. By allowing oxygen to completely suffuse the nasal cavity during exhalation, breathing with the mouth open may enable more efficient CO_2_ washout and provide a larger anatomic reservoir. With inhalation, nasal oxygen is entrained and contributes to higher F_I_O_2_.

### PEEP effect

Although HFNC is an open system, high flow from the nasal cannula prevails against some of the resistance of expiratory flow and increases airway pressure. In an *in vitro* study with and without a pressure-limiting valve to limit airway pressure, a neonate model of HFNC showed that airway pressure increased as flow increased [13]. *In vivo* results from the same observational study indicated that, when there is escape of gas, end-expiratory esophageal pressure does not increase at 3 cmH_2_O. Parke et al. measured nasopharyngeal pressure in post-cardiac surgery patients [14]. Comparing HFNC and face mask delivery, at 35 L/min flow, while HFNC nasopharyngeal pressure increased to 2.7 ± 1.04 cmH_2_O with the mouth closed and 1.2 ± 0.76 cmH_2_O with the mouth open, it was around zero with the face mask. Affected by gender, body mass index (BMI), the mouth closed or opened, and flow, other authors have also reported positive pharyngeal pressure with HFNC [10,15-18]. With the mouth closed, pharyngeal pressure increases as flow increases. With the mouth open, even at 60 L/min flow, pharyngeal pressure remained below 3 cmH_2_O [14]. In postoperative patients, as inspiratory flow increased, airway pressure increased: 1.52 ± 0.7, 2.21 ± 0.8, and 3.1 ± 1.2 cmH_2_O at 40, 50, and 60 L/min of flow, respectively [15].

In several studies that reported increased pharyngeal pressure with HFNC, it was unclear whether HFNC actually increases lung volume or recruits collapsed alveoli. Corley et al. evaluated end-expiratory lung volume using electrical lung impedance tomography and found that end-expiratory lung volume was greater with HFNC than with low-flow oxygen therapy [10]. In addition, the effect was more pronounced in patients with higher BMI. Riera et al. also measured, in supine and in prone postures, end-expiratory lung volume by electrical lung impedance tomography [18]. It was greater in either position with HFNC. Mean upper airway pressure with the mouth closed showed increasing pressure with increasing delivered gas flow [7].

### Fraction of inspired oxygen

Actual F_I_O_2_ is not stable with low-flow oxygen delivery and generally much lower than equipment algorithm predicts [19,20]. At 1–6 L/min, F_I_O_2_ ranged from 0.26 to 0.54 during calm breathing and 0.24 to 0.45 during rapid breathing [12], increasing to, respectively, 0.54–0.75 and 0.49–0.72 at 6–15 L/min. F_I_O_2_ was higher during mouth-open breathing than during mouth-closed breathing. With HFNC, especially at high flow, actual F_I_O_2_ was close to calculated (predicted) F_I_O_2_. Ritchie et al. performed hypopharyngeal oxygraphy, capnography, and measurement of pressure [15]. During nose breathing at rest, above 30 L/min, the measured F_I_O_2_ was close to the delivered F_I_O_2_. High peak inspiratory flow with exercise was associated with increased air entrainment resulting in lower F_I_O_2_.

### Humidification

In clinical settings, there are situations in which air moisture is reduced, for example, when gas, such as piped oxygen, is delivered from an artificial flow source or when an endotracheal or tracheostomy tube bypasses the upper airway, where most humidification would naturally occur. Conventional oxygen devices delivering dry and unwarmed gas are associated with mask discomfort, nasal dryness, oral dryness, eye irritation, nasal and eye trauma, gastric distension, and aspiration [3,21,22]. Gas, when unwarmed and dry, may have a variety of untoward effects on patients with respiratory support. It is well known that cold air induces bronchoconstriction [23,24]. Greenspan et al. have demonstrated that as little as 5 min of ambient gas delivered directly at the trachea can cause a significant decrease in pulmonary compliance and conductance in infants [25]. By contrast, adequately conditioned gas has less impact on the physiological response of the lungs. Saslow et al. have found greater respiratory compliance in infants with 5 L/min of high-flow delivery with conditioned gas compared to 6 cmH_2_O of conventional CPAP using a standard humidification unit [26]. Conditioning of the gas minimizes airway constriction and reduces the work of breathing. Furthermore, conditioned gas improves mucociliary function [27], facilitates clearance of secretions, and is associated with less atelectasis, resulting in a good ventilation/perfusion ratio and better oxygenation. In other words, conditioning gases results in more effective delivery of oxygen to the lungs. It can be particularly important for patients with secretion problems, such as those with COPD.

NIV delivers medical gas at high flow; if these gases are inadequately humidified, oral dryness and patient discomfort are likely [28]. Oto et al. have carried out serial measurements during 24 h of absolute humidity (AH) inside the oronasal masks of subjects undergoing NIV for acute respiratory failure (ARF) [29]. Sixteen subjects were enrolled, and AH inside the mask was 30.0 ± 2.6 mg H_2_O/L (range 23.1–33.3 mg H_2_O/L). The absolute humidity of gas delivered to oronasal masks during NIV, affected by humidifier settings and the amount of leakage, varied among patients at equivalent humidifier settings. Since HFNC delivers medical gas at up to 60 L/min flow, inadequate humidification may cause the same untoward effects as NIV. Chanques et al. have shown that while bubble humidifiers deliver poor levels of humidity and are associated with significant discomfort, a heated humidifier was associated with a decrease of dryness [3]. While humidification is greatly determined by the performance of humidifying devices, it is also affected by patient breathing patterns. Evaluating the performance of popular HFNC systems, Chikata et al. found that, regardless of patient V_T_ or minute volume, they delivered adequately warmed and humidified gas when gas flow was greater than 40 L/min [30]. For intensive care unit (ICU) patients with ARF, it is unusual for HFNC to be interrupted owing to discomfort. In another study in which HFNC was used for an average of 2.8 ± 1.8 days (max. 7 days), intolerance did not cause HFNC to be discontinued and no unexpected side effects were detected. The evidence suggests that HFNC can be regarded as a very comfortable gas delivery system. Provision of essential humidity through HFNC can prevent drying of the airway, avoiding the inflammatory response caused by the drying of the mucosa.

### Clinical trials

#### Hypercapnic respiratory failure

Hypercapnic respiratory failure is a frequently encountered problem [31]. Patients with this condition present a significant challenge to respiratory and critical care services, as many are unsuitable for mechanical ventilation and most have multiple comorbidities; more or less by default, NIV has become established as the primary modality for respiratory support for these patients [32]. Because of poor mask tolerance, however, it is inapplicable to some patients [33,34]. Millar et al. have reported the successful use of HFNC oxygen therapy to manage the hypercapnic respiratory failure of a patient unable to tolerate conventional NIV [35]. Bräunlich evaluated the effect of HFNC in healthy volunteers, COPD patients, and idiopathic pulmonary fibrosis (IPF) patients [36]. Compared with unaided breathing, V_T_ increased in the COPD and IPF groups, while it decreased in the healthy volunteers. Breathing frequency and minute volume decreased in all groups. Nilius et al. investigated the effects of HFNC on COPD patients with chronic hypercapnic respiratory failure [37]. After receiving 20 L/min of room air and 2 L/min of oxygen for 45 min through a nasal cannula either into both nostrils or into one nostril, individual responses to HFNC varied, but breathing frequency decreased for some and PaCO_2_ decreased for some. Testing COPD patient exercise breathing with an unloaded bicycle ergometer, Chatila et al. observed increased exercise capacity with improved oxygenation with HFNC compared to spontaneous breathing [38]. There is much evidence to suggest that HFNC is a highly promising treatment for therapy for some types of hypercapnic respiratory failure.

#### Hypoxemic respiratory failure

Maintaining adequate oxygenation depends on properly managing F_I_O_2_ and PEEP. Oxygen is generally provided via a face mask or nasal cannula, and oxygen delivery is limited to no more than 15 L/min. Using conventional methods, when there are large differences between patient inspiratory flow and delivered flow, F_I_O_2_ values are difficult to control and are usually lower than calculation predicts. HFNC, however, does literally deliver high flow and actual F_I_O_2_ values are usually close to delivered F_I_O_2_ [15].

For patients with hypoxemic respiratory failure, how well does HFNC work in maintaining stable F_I_O_2_ and positive pharyngeal pressure? From the reported physiological effects of HFNC, high flow through a nasal cannula meets resistance from patient expiration, and pressure in the pharynx increases. Since the cannula is part of an open system, pharyngeal pressure may not be high enough comparing to NIV or invasive mechanical ventilation [14,15].

HFNC has been found to be effective for mild to moderate hypoxemic respiratory failure. Sztrymf et al. investigated the efficiency, safety, and outcome of HFNC in ICU patients with ARF [7]. Patients (38 in total) were enrolled when they either required more than 9 L/min of oxygen to achieve a SpO_2_ > 92% or exhibited persistent signs of respiratory distress. Oxygen flow of about 15 L/min via a face mask was replaced with HFNC of 49 ± 9 L/min. HFNC was associated with significant reductions in breathing frequency, heart rate, dyspnea score, supraclavicular retraction and thoracoabdominal asynchrony, and significant improvement in SpO_2_. The duration of HFNC was 2.8 ± 1.8 days (max. 7 days), and HFNC was not stopped because of intolerance. In another study, Sztrymf et al. investigated the effects of HFNC on alleviating respiratory distress and ameliorating oxygenation in adult ICU patients with mild to moderate hypoxemic ARF [8]. The etiology of ARF was mainly community-acquired pneumonia and sepsis. Oxygen flow of 15 L/min via a face mask was changed to HFNC of 40 L/min. Under HFNC, breathing frequency decreased and oxygenation improved. After a median delay of 17.5 h of HFNC therapy, 6 of 20 (30%) were subsequently intubated owing to septic shock, gastrointestinal hemorrhage, and worsening pneumonia. In a cardiothoracic ICU, Parke et al. evaluated whether HFNC was better tolerated, with fewer treatment failures, than conventional face mask delivery in patients with mild to moderate hypoxemic respiratory failure [17]. For the HFNC group, flow was 35 L/min, and F_I_O_2_ were titrated to maintain SpO_2_ at ≥95%: therapy failed for 3 of 29 (10%) in the HFNC group and 12 of 27 (44%) in the conventional group. Roca et al. have also reported good tolerance to HFNC in patients with ARF [9]. HFNC has also been applied in emergency departments, where it was found to alleviate dyspnea and improve oxygenation in patients with hypoxemic ARF [39,40]. These reports have demonstrated how effective HFNC can be as a first-line treatment for ICU patients with ARF.

On the other hand, HFNC has not been recommended for severe hypoxemic respiratory failure because of doubts about ensuring positive pharyngeal pressure. And there have been few reports of using HFNC oxygen therapy for severe acute respiratory infection (SARI). Rello et al. applied HFNC to acute hypoxemic respiratory failure due to influenza A/H1N1 [41]. Of 35 patients, 5 patients were treated with conventional oxygen therapy and 10 needed immediate intubation. The remaining 20 patients were unable to maintain SpO_2_ above 92% with supplemental oxygen greater than 9 L/min: 9 were successfully treated with HFNC and intubation was avoided and the 11 others were subsequently intubated. Prospectively observing ARDS patients, Messika et al. evaluated the indications and effects of HFNC [42]. HFNC was applied in 45 acute respiratory distress syndrome (ARDS) patients as the first-line treatment: 40% of the patients were subsequently intubated, HFNC failure being associated with high SAPS II scores. Even without measurement of PEEP (CPAP) in noninvasive HFNC, although it has some limitations, the study suggests that HFNC oxygen therapy is a promising modality for the early treatment of adults with severe ARF. With the proviso that further study is needed, these studies suggest that HFNC delivery could be effective during severe hypoxemic ARF. The kind of rigorous evidence we need to confidently guide clinical choices will be provided by the FLORALI (high FLow Oxygen therapy for Resuscitation of patients with Acute Lung Injury) study, which has completed patient enrollment [43].

#### Post-extubation

Re-intubation is associated with increased ICU and in-hospital length of stay and mortality [44,45]. HFNC seems to reduce the need for noninvasive positive pressure ventilation (NPPV) and re-intubation. Maggiore et al. have compared the effects of delivery via a Venturi mask and HFNC on oxygenation and clinical outcomes [46]. The PaO_2_/F_I_O_2_ ratio was higher with HFNC than with the Venturi mask. With HFNC, fewer patients required NPPV and re-intubation. Meanwhile, Parke et al. randomized patients after cardiac surgery to HFNC (45 L/min) or conventional delivery [47]. Oxygen therapy was started after extubation and continued to day 2. No difference in oxygenation was found between the groups, and PaCO_2_ was lower in the HFNC group at 4 h post-extubation and the next morning. Significantly fewer patients required escalation of respiratory support in the HFNC group. Similarly, Tiruvoipati et al. compared HFNC and high-flow face mask delivery [48]. For extubated patients, no differences in respiratory and hemodynamic parameters were found between modes, but tolerance of HFNC was better. For the kind of rigorous evidence we need to confidently guide clinical choices, we await the findings of the OPERA trial, which is evaluating how well HFNC prevents post-extubation hypoxemia after abdominal surgery [49].

#### Pre-intubation oxygenation

Intubation in the ICU is often performed for hypoxemic, unstable patients and is associated with significant complications [50,51]. Before tracheal intubation, to enhance oxygenation, NIV can be applied [52]. If it is, when the mask has to be removed during laryngoscopy, the patient is deprived of oxygen during the procedure. Because nasal cannulas do not interfere with the laryngoscopy, HFNC could be used to deliver oxygen during the apneic period of tracheal intubation. A recent animal study has elegantly demonstrated significantly delayed occurrence of severe desaturation during apnea when direct pharyngeal administration of 10 L/min oxygen was carried out during intubation of hypoxemic piglets [11]. In a clinical trial, enrolling 101 patients, Miguel-Montanes et al. compared the pre- and per-procedure oxygenation of ventilation using a nonrebreathing bag reservoir face mask and ventilation using HFNC during tracheal intubation of ICU patients [53]. With the nonrebreathing bag reservoir face mask, the median lowest SpO_2_ during intubation was 94%, and with HFNC, it was 100%. The authors concluded that HFNC significantly reduced the prevalence of severe hypoxemia and that its use could improve patient safety during intubation in ICU.

While it is clear that the use of HFNC delivery during intubation of ICU patients should be further evaluated in clinical studies, for ethical reasons, a randomized controlled trial may not be the best type of study. Given the preponderance of published data clearly showing superior oxygenation by HFNC delivery, a randomized controlled comparison with face mask delivery could be judged to create avoidable risk for patients in the face mask group.

#### Sleep apnea

Obstructive sleep apnea (OSA) is attributed to upper airway collapse that is associated with intermittent hypoxemia, neurocognitive dysfunction, and cardiovascular morbidity [54-56]. The treatment of sleep apnea includes medical and surgical options. While CPAP is the most effective treatment, adherence is suboptimal and a large number of patients are left untreated [57]. McGinley et al. found that HFNC delivery for OSA alleviated upper airway obstruction [58,59]. In both children and adults, HFNC with 20 L/min of flow was applied. In children, this reduced the amount of inspiratory flow limitation and decreased arousals and the apnea-hypopnea index. HFNC also reduced arousals and the apnea-hypopnea index in adults.

Disordered breathing during sleep is also common among acute stroke patients and is associated with neurologic worsening and poor outcome. Although CPAP is effective in treating sleep disordered breathing, with stroke patients, it is often abandoned owing to patient discomfort. It has been reported that HFNC (18 L/min) was well tolerated and decreased the apnea-hypopnea index and the oxygen desaturation index [60]. The percentage of slow-wave sleep significantly increased, and quality of sleep was better. HFNC therapy is viable for acute stroke patients.

#### Acute heart failure

Various oxygenation methods are used for treating respiratory failure occurring with acute heart failure [61]. Sometimes, after patients have been stabilized by emergency methods, a degree of dyspnea or hypoxemia remains. HFNC is a good alternative means of supplementing oxygenation. Carratalá Perales et al. studied the effect of HFNC on patients with dyspnea and hypoxemia following NIV [62]. Successfully treated with HFNC, all five patients showed clinical improvement. Moriyama et al. successfully maintained oxygenation in a patient with life-threatening reperfusion pulmonary edema, which arose after transluminal pulmonary angioplasty, by applying, for 3 days, HFNC at F_I_O_2_ 1.0 and 50 L/min of flow [63].

#### Others

Hypoxemia is common during invasive procedures, and supplemental oxygen may be delivered by various interfaces. Lucangelo et al. used HFNC during bronchoscopy in adults and compared the effects of HFNC at 40 and 60 L/min with 40 L/min delivered via a Venturi mask [64]. At the end of the procedure, HFNC at 60 L/min resulted in better oxygenation than 40 L/min delivered either by a Venturi mask or by HFNC. Oxygenation was also better at 10 min after the completion of the procedure. In a case reported by Diab et al., for an orthotropic lung transplant recipient who required diagnostic bronchoscopy, HFNC effectively prevented hypoxemia [65].

Patients with do-not-intubate (DNI) status and respiratory failure are generally treated with NIV [66,67], which has been found effective in relieving sensations of dyspnea. HFNC may be an effective alternative to NIV. Peters et al. assessed the efficacy of HFNC in DNI patients with hypoxemic respiratory distress [68]. The mean age was 73 years, and the underlying diseases were pulmonary fibrosis, pneumonia, COPD, cancer, hematologic malignancy, and congestive heart failure. Only 9 of 50 patients were escalated to NIV, and 82% were maintained on HFNC. The median duration of HFNC was 30 h. HFNC can provide adequate oxygenation for patients with hypoxemic respiratory failure and may be an alternative to NIV for DNI patients.

Many clinical reports of HFNC have been published. Díaz-Lobato et al. treated ARF of neuromuscular origin [69], and Boyer et al. treated pulmonary fibrosis for more than 30 days [70]. Generally, over the long term, it is not possible to continuously support respiration with NIV. Byerly et al. reported successful using HFNC to treat a pediatric patient with inhalation injury, post-extubation stridor, and a high risk of extubation failure [71]. Calvano et al. applied HFNC to a 92-year-old woman with delirium and dementia who was in the ICU for multi-lobar pneumonia with severe hypoxemia [72]. After she had rejected various facial and nasal masks, it was found that she could tolerate HFNC. It reduced her agitation, ameliorated her dyspnea, improved oxygenation, and increased her comfort at the end of life.

### Contraindication

HFNC gains more and more attention, and physicians apply it to patients with a variety of diseases and a variety of conditions. There have never been reported any big randomized clinical trials and no strong evidence of the clinical application of HFNC and conversely no absolute contraindications. We should be careful to apply it to patients to whom NPPV is contraindicated. Table 1 shows contraindications of NPPV. It is an open system and we do not have to care about the tight contact of interfaces, and HFNC can be applicable to patients with claustrophobia.

**Table 1:** **Contraindication of noninvasive positive pressure ventilation**

	**Contraindication**
1.	Consciousness disorder
	a. No response
	b. Agitated
	c. Uncooperative
2.	Claustrophobia
3.	Airway obstruction
4.	Facial injury, facial malformation
5.	A lot of sputum
6.	Risk of aspiration
7.	Unstable hemodynamics
	a. Shock
	b. Intractable arrhythmia
	c. Post-CPR
8.	Respiratory arrest

## Conclusions

HFNC oxygen delivery is proving to be a valuable aid and has been gaining attention as an alternative means of respiratory support for critically ill patients. Physicians have been using it for patients with a variety of underlying diseases. It seems to be effective for treating hypercapnic respiratory failure and mild to moderate hypoxemic respiratory failure. Some important issues remain to be resolved, however, such as the indication of HFNC and criteria for timing the start of HFNC, for stopping HFNC, and for escalating treatment. Since HFNC is noninvasive, the PEEP (CPAP) level is not measured. Despite these issues, a growing body of evidence suggests that HFNC oxygen therapy is an innovative and effective modality for the early treatment of adults with respiratory failure associated with diverse underlying diseases.

## References

[1] Girou E, Brun-Buisson C, Taillé S, Lemaire F, Brochard L (2003). Secular trends in nosocomial infections and mortality associated with noninvasive ventilation in patients with exacerbation of COPD and pulmonary edema. JAMA.

[2] Campbell EJ, Baker MD, Crites-Silver P (1988). Subjective effects of humidification of oxygen for delivery by nasal cannula. A prospective study. Chest.

[3] Chanques G, Contantin JM, Sauter M, Jung B, Sebbane M, Verzilli D (2009). Discomfort associated with underhumidified high-flow oxygen therapy in critically ill patients. Intensive Care Med.

[4] Mayfield S, Jauncey-Cook J, Hough JL, Schibler A, Gibbons K, Bogossian F (2014). High-flow nasal cannula therapy for respiratory support in children. Cochrane Database Syst Rev.

[5] Dani C, Pratesi S, Migliori C, Bertini G (2009). High flow nasal cannula therapy as respiratory support in the preterm infant. Pediatr Pulmonol.

[6] Itagaki T, Okuda N, Tsunano Y, Kohata H, Nakataki E, Onodera M (2014). Effect of high-flow nasal cannula on thoraco-abdominal synchrony in adult critically ill patients. Respir Care.

[7] Sztrymf B, Messika J, Bertrand F, Hurel D, Leon R, Dreyfuss D (2011). Beneficial effects of humidified high flow nasal oxygen in critical care patients: a prospective pilot study. Intensive Care Med.

[8] Sztrymf B, Messika J, Mayot T, Lenglet H, Dreyfuss D, Ricard J-D (2012). Impact of high-flow nasal cannula oxygen therapy on intensive care unit patients with acute respiratory failure: a prospective observational study. J Crit Care.

[9] Roca O, Riera J, Torres F, Masclans JR (2010). High-flow oxygen therapy in acute respiratory failure. Respir Care.

[10] Corley A, Caruana LR, Barnett AG, Tronstad O, Fraser JF (2011). Oxygen delivery through high-flow nasal cannulae increase end-expiratory lung volume and reduce respiratory rate in post-cardiac surgical patients. Br J Anaesth.

[11] Frizzola M, Miller TL, Rodriguez ME, Zhu Y, Rojas J, Hesek A (2011). High-flow nasal cannula: impact on oxygenation and ventilation in an acute lung model. Pediatr Pulmonol.

[12] Wettstein RB, Shelledy DC, Peters JI (2005). Delivered oxygen concentrations using low-flow and high-flow nasal cannulas. Respir Care.

[13] Lampland AL, Plumm B, Meyers PA, Worwa CT, Mammel MC (2009). Observational study of humidified high-flow nasal cannula compared with nasal continuous positive airway pressure. J Pediatr.

[14] Parke R, McGunness S, Eccleston M (2009). Nasal high-flow therapy delivers low level positive airway pressure. Br J Anaesth.

[15] Ritchie JE, Williams AB, Gerard C, Hockey H (2011). Evaluation of a humidified nasal high-flow oxygen system, using oxygraphy, capnography and measurement of upper airway pressures. Anaesth Intensive Care.

[16] Groves N, Tobin A (2007). High flow nasal oxygen generates positive airway pressure in adult volunteers. Aust Crit Care.

[17] Parke RL, McGuinness SP (2013). Pressures delivered by nasal high flow oxygen during all phases of the respiratory cycle. Respir Care.

[18] Riera J, Pérez P, Cortés J, Roca O, Masclans JR, Rello J (2013). Effect of high-flow nasal cannula and body position on end-expiratory lung volume: a cohort study using electrical impedance tomography. Respir Care.

[19] Bazuaye EA, Stone TN, Corris PA, Gibson GJ (1992). Variability of inspired oxygen concentration with nasal cannulas. Thorax.

[20] Markovitz GH, Colthurst J, Storer TW, Cooper CB (2010). Effective inspired oxygen concentration measured via transtracheal and oral gas analysis. Respir Care.

[21] Andres D, Thurston N, Brant R, Flemons W, Fofonoff D, Ruttimann A (1997). Randomized double-blind trial of the effects of humidified compared with nonhumidified low flow oxygen therapy on the symptoms of patients. Can Respir J.

[22] Campbell EJ, Baker MD, Crites-Silver P (1988). Subjective effects of humidification of oxygen for delivery by nasal cannula. A prospective study. Chest.

[23] Berk JL, Lenner KA, McFadden ER (1987). Cold-induced bronchoconstriction: role of cutaneous reflexes vs direct airway effects. J Appl Physiol.

[24] Fontanari P, Burnet H, Zattara-Hartmann MC, Jammes Y (1996). Changes in airway resistance induced by nasal inhalation of cold dry, dry, or moist air in normal individuals. J Appl Physiol.

[25] Greenspan JS, Wolfson MR, Shaffer TH (1991). Airway responsiveness to low inspired gas temperature in preterm neonates. J Pediatr.

[26] Saslow JG, Aghai ZH, Nakhla TA, Hart JJ, Lawrysh R, Stahl GE (2006). Work of breathing using high-flow nasal cannula in preterm infants. J Perinatol.

[27] Salah B, Dinh Xuan AT, Fouilladieu JL, Lockhart A, Regnard J (1988). Nasal mucociliary transport in healthy subjects is slower when breathing dry air. Eur Respir J.

[28] Oto J, Nakataki E, Okuda N, Onodera M, Imanaka H, Nishimura M (2014). Hygrometric properties of inspired gas and oral dryness in patients with acute respiratory failure during noninvasive ventilation. Respir Care.

[29] Oto J, Imanaka H, Nishimura M (2011). Clinical factors affecting inspired gas humidification and oral dryness during noninvasive ventilation. J Crit Care.

[30] Chikata Y, Izawa M, Okuda N, Itagaki T, Nakataki E, Onodera M (2014). Humidification performances of two high flow nasal cannula devices: a bench study. Respir Care.

[31] Dewan NA, Bell CW (1994). Effect of low flow and high flow oxygen delivery on exercise tolerance and sensation of dyspnea. A study comparing the transtracheal catheter and a nasal prongs. Chest.

[32] Brochard L, Mancebo J, Wysocki M, Lofaso F, Conti G, Rauss A (1995). Noninvasive ventilation for acute exacerbations of chronic obstructive pulmonary disease. N Engl J Med.

[33] Ozyilmaz E, Ozsancak A, Nava S (2014). Timing of noninvasive ventilation failure: causes, risk factors, and potential remedies. BMC Pulm Med.

[34] Nicolini A, Ferrera L, Santo M, Ferrari-Bravo M, Del Forno M, Sclifò F (2014). Noninvasive ventilation for hypercapnic exacerbation of chronic obstructive pulmonary disease: factors related to noninvasive ventilation failure. Pol Arch Med Wewn.

[35] Millar J, Lutton S, O’Connor P (2014). The use of high-flow nasal oxygen therapy in the management of hypercarbic respiratory failure. Ther Adv Respir Dis.

[36] Bräunlich J, Beyer D, Mai D, Hammerschmidt S, Seyfarth H-J, Wirtz H (2013). Effects of nasal high flow on ventilation in volunteers, COPD and idiopathic pulmonary fibrosis patients. Respiration.

[37] Nilius G, Franke K-J, Domanski U, Rühle K-H, Kirkness JP, Schneider H (2013). Effects of nasal insufflation on arterial gas exchange and breathing pattern in patients with chronic obstructive pulmonary disease and hypercapnic respiratory failure. Adv Exp Med Biol.

[38] Chatila W, Nugent T, Vance G, Gaughan J, Criner GJ (2004). The effects of high-flow vs low-flow oxygen on exercise in advanced obstructive airways disease. Chest.

[39] Kelly GS, Simon HK, Sturm JJ (2013). High-flow nasal cannula use in children with respiratory distress in the emergency department: predicting the need for subsequent intubation. Pediatr Emerg Care.

[40] Lenglet H, Sztrymf B, Leroy C, Brun P, Deyfuss D, Ricard J-D (2012). Humidified high flow nasal oxygen during respiratory failure in the emergency department: feasibility and efficacy. Respir Care.

[41] Rello J, Pérez M, Roca O, Poulakou G, Souto J, Laborda C (2012). High-flow nasal therapy in adults with severe acute respiratory infection. A cohort study in patients with 2009 influenza A/H1N1v. J Crit Care.

[42] Messika J, Ahmed KB, Gaudry S, Miguel-Montanes R, Rafat C, Sztrymf B (2015). Use of high-flow nasal cannula oxygen therapy in subjects with ARDS: a 1-year observational study. Respir Care.

[43] Poitiers University Hospital. Clinical effect of the association of noninvasive ventilation and high flow nasal oxygen therapy in resuscitation of patients with acute lung injury (FLORALI Study). http://clinicaltrials.gov/ct2/show/record/NCT01320384

[44] Torres A, Gatell JM, Aznar E, El-Ebiary M, Puig de la Bellacasa J, González J (1995). Re-intubation increases the risk of nosocomial pneumonia in patients with needing mechanical ventilation. Am J Respir Crit Care Med.

[45] Esteban A, Anzueto A, Frutos F, Alía I, Brochard L, Stewart TE (2002). Characteristics and outcomes in adult patients receiving mechanical ventilation. A 28-day international study. JAMA.

[46] Maggiore SM, Idone FA, Vaschetto R, Festa R, Cataldo A, Antonicelli F (2014). Nasal high-flow versus Venturi mask oxygen therapy after extubation. Am J Respir Crit Care Med.

[47] Parke R, McGuinness S, Dixon R, Jull A (2013). Open-label, phase II study of routine high-flow nasal oxygen therapy in cardiac surgical patients. Br J Anaesth.

[48] Tiruvoipati R, Lewis D, Haji K, Botha J (2010). High-flow nasal oxygen vs high-flow face mask: a randomized crossover trial in extubated patients. J Crit Care.

[49] Futier E, Paugam-Burtz C, Constantin J-M, Pereira B, Jaber S (2013). The OPERA trial – comparison of early nasal high flow oxygen therapy with standard care for prevention of postoperative hypoxemia after abdominal surgery: study protocol for a multicenter randomized controlled trial. Trials.

[50] Schwartz DE, Matthay MA, Cohen NH (1995). Death and other complications of emergency airway management in critically ill adults. A prospective investigation of 297 tracheal intubations. Anesthesiology.

[51] Mort TC (2004). Emergency tracheal intubation: complications associated with repeated laryngoscopic attempts. Anesth Analg.

[52] Baillard C, Fosse JP, Sebbane M (2006). Noninvasive ventilation improves preoxygenation before intubation of hypoxic patients. Am J Respir Crit Care Med.

[53] Miguel-Montanes R, Hajage D, Messika J, Bertrand F, Gaudry S, Rafat C (2015). Use of high-flow nasal cannula oxygen therapy to prevent desaturation during tracheal intubation of intensive care patients with mild-to-moderate hypoxemia. Crit Care Med.

[54] Cörtük M, Akyol S, Baykan AO, Kiraz K, Uçar H, Cayli M, Kandiş H. Aortic stiffness increases in proportion to the severity of apnea-hypopnea index in patients with obstructive sleep apnea syndrome. Clin Respir J. 2014. doi:10.1111/crj.12244 10.1111/crj.12244 25401950

[55] Sahlin C, Sandberg O, Gustafson Y, Bucht G, Carlberg B, Stenlund H (2008). Obstructive sleep apnea is a risk factor for death in patients with stroke. A 10-year follow-up. Arch Intern Med.

[56] Fonseca MI, Pereira T, Caseiro P (2014). Death and disability in patients with sleep apnea – a meta-analysis. Arq Bras Cardiol.

[57] Marcus CL, Rosen G, Ward S, Halbower AC, Sterni L, Lutz J (2006). Adherence to and effectiveness of positive airway pressure therapy in children with obstructive sleep apnea. Pediatrics.

[58] McGinley BM, Patil SP, Kirkness JP, Smith PL, Schwartz AR, Schneider H (2007). A nasal cannula can be used to treat obstructive sleep apnea. Am J Respir Crit Care Med.

[59] McGinley B, Halbower A, Schwartz AR, Smith PL, Patil SP, Schneider H (2009). Effect of a high-flow open nasal cannula system on obstructive sleep apnea in children. Pediatrics.

[60] Haba-Rubio J, Andries D, Rey V, Michel P, Tafti M, Heinzer R (2012). Effect of transnasal insufflation on sleep disordered breathing in acute stroke: a preliminary study. Sleep Breath.

[61] Masip J, Betbesé AJ, Páez J, Vecilla F, Cañizares R, Padró J (2000). Non-invasive pressure support ventilation versus conventional oxygen therapy in acute cardiogenic pulmonary oedema: a randomized trial. Lancet.

[62] Carratalá Perales JM, Llorens P, Brouzet B, Albert Jiménez AR, Fernández-Cañadas JM, Carbajosa Dalmau J (2011). High-flow therapy via nasal cannula in acute heart failure. Rev Esp Cardiol.

[63] Moriyama K, Satoh T, Motoyasu A, Kohyama T, Kotani M, Kanai R, et al. High-flow nasal cannula therapy in a patient with reperfusion pulmonary edema following percutaneous transluminal pulmonary angioplasty. Case Rep Pulmonol 2014. doi.org/1 k0.115/2014/837612. 10.1155/2014/837612 PMC4122149 25126437

[64] Lucangelo U, Vassallo FG, Marras E, Ferluga M, Beziza E, Comuzzi L (2012). High-flow nasal interface improves oxygenation in patients undergoing bronchoscopy. Crit Care Res Pract.

[65] Diab S, Fraser JF (2014). Maintaining oxygenation successfully with high flow nasal cannula during diagnostic bronchoscopy on a postoperative lung transplant patient in the intensive care. Case Rep Crit Care.

[66] Schettino G, Altobelli N, Kacmarek RM (2005). Noninvasive positive pressure ventilation reverses acute respiratory failure in select “do-not-intubate” patients. Crit Care Med.

[67] Levy M, Tanios MA, Nelson D, Short K, Senechia A, Vespia J (2004). Outcomes of patients with do-not-intubate orders treated with noninvasive ventilation. Crit Care Med.

[68] Peters SG, Holets SR, Gay PC (2013). High-flow nasal cannula therapy in do-not-intubate patients with hypoxemic respiratory distress. Respir Care.

[69] Díaz-Lobato S, Folgado MA, Chapa A, Alises SM (2013). Efficacy of high-flow oxygen by nasal cannula with active humidification in a patient with acute respiratory failure of neuromuscular origin. Respir Care.

[70] Boyer A, Vargas F, Delacre M, Saint-Léger M, Clouzeau B, Hilbert G (2011). Prognostic impact of high-flow nasal cannula oxygen supply in an ICUY patient with pulmonary fibrosis complicated by acute respiratory failure. Intensive Care Med.

[71] Byerly FL, Haithcock JA, Buchanan IB, Short KA, Cairns BA (2006). Use of high flow nasal cannula on a pediatric burn patient with inhalation injury and post-extubation stridor. Burns.

[72] Calvano TP, Sill JM, Kemp KR, Chung KK (2008). Use of high-flow oxygen delivery system in a critically ill patient with dementia. Respir Care.

